# Thank you very much!

**DOI:** 10.1590/2177-6709.22.6.009-010.edt

**Published:** 2017

**Authors:** David Normando

**Affiliations:** 1 Adjunct professor, Universidade Federal do Pará (UFPA), School of Dentistry, Belém, Pará, Brazil. Coordinator, Universidade Federal do Pará (UFPA), Graduate program in Dentistry, and ABO-Pará, Specialization course in Orthodontics, Belém, Pará, Brazil.

*“Happy people remember the past with gratitude, rejoice in the present and face the future without fear.”*

Epicurus, Greek philosopher

For the past 6 years, I have been editor-in-chief of this journal, and this will be my last text in this position. Editorials are free texts, and after 33 of them focused on discussing scientific methodologies, explaining the need for changes^1^ ^-^ ^3^ and promoting the growth of this journal^4^ and of Brazilian Orthodontics,^5^ I ask our readers to let me write a personal text. Gratitude is a reliable and precious asset.

My story with the *Dental Press Journal of Orthodontics* (DPJO) began as a reader in the late 1990s. After engaging as an author in 2005, I was invited by Prof Adilson Ramos to become a reviewer. In a country where titles are worth more than knowledge itself, I eloquently give thanks to that human being who has acknowledged the merits of someone so far from the development axis of the country. In 2010, even without a PhD degree, I was invited to work as associate editor by the, at that time, editor-in-chief, Jorge Faber. I knew him little in person, almost nothing, but the already existing admiration reverberated through recognition, in the absence of personal issues. The following year I received Faber’s invitation to become editor-in-chief of DPJO. The recognition of these two colleagues, in a country marked by the lack of attention to competence, made me enthusiastically believe in this project.

However, none of the movements or beliefs of my predecessors would have been possible without the acquiescence of the couple who dreamed about the *Dental Press Publishing*. I am convinced that doubts must have arisen when considering if this professional from the remote Amazon could captain such an important achievement of Brazilian Orthodontics. Many thanks to Drs Laurindo & Teresa Furquim, for believing without prejudice. Brazilian Orthodontics may not even know the debt of gratitude they owe to your work. I know even less.

Frustration and fear of failure are neuralgic burdens that slip into the imaginary of the first step of a route to be drawn. Even harder to preserve such an acclaimed and applauded pathway. With that sentence impregnated in my mind, at the beginning of my journey as an editor I established goals that I have published throughout my editorials. With great pride, examining the past, I note that all of them have been effectively achieved, perhaps even overfulfilled. The time between the article’s submission and its actual publication was reduced to a maximum of 12 months;^3^ we were indexed in PubMed,^6^ the most important bibliographic database in health sciences; we quadrupled the scientific impact of the journal on the SCImago database;^7^ and conquered an increase in Qualis/Capes* evaluation within the dentistry area in Brazil.^7^ We are today the fifth orthodontic journal in total number of citations (*cites per doc*/3 years) in the world and, among the Brazilian scientific journals, we jumped from 162^nd^ position in the SJR (*SCImago Journal & Country Rank*) in 2012 to 37^th^ in 2017. Furthermore, we are the second among the Brazilian dental journals.

Obviously, these are not personal achievements - oppositely to this editorial -, but are result of the predecessors’ efforts to give continuity to this work; the efficient staff of Dental Press publishing house; the researchers who relied on this journal, reviewers, and a group of brilliant associate editors. Imagine how easy it is to work with so many brilliant professionals as Carlos Flores-Mir (University of Alberta), Daniela Garib (USP), Fernanda Angelieri (University of Guarulhos), Flavia Artese (UERJ), Ildeu Andrade (PUC-MG), Leandro Marques (UFVJM), Luciane Menezes (PUC-RS), Matheus Pithon (UESB), Renato Martins (UNESP) and Telma Martins (UFBA). Believing is a primal attitude, so I thank you for accepting me as your helmsman.

Add to that researchers from one of the most valued Orthodontics in the world. Crazy people, who believe they can produce cutting-edge scientific knowledge in a land of scarce resources. As usual, every two years, we thank researchers who entrusted their manuscripts to publication in the *Dental Press Journal of Orthodontics*. Among them we highlight those who published three or more original articles during the 2016-2017 biennium: Attyia Shaikh, André Machado, Camilla Vieira, Carlos Flores-Mir, Célia Pinzan-Vercelino, David Normando, Eduardo Sant’Anna, Guilherme Janson, Jonas Capelli Jr., José Fernando Castanha Henriques, Júlio Gurgel, Leopoldino Capelozza Filho, Marcel Farret, Mubassar Fida, Renato Martins. Our acknowledgments and many thanks.

For the current biennium, we have had the collaboration of authors from 81 Brazilian institutions and 22 foreign institutions (Germany, Belgium, China, Canada, Colombia, South Korea, Denmark, Egypt, USA, Holland, Iran, India, Italy, Japan, Pakistan, Paraguay, Peru, Portugal, United Kingdom, Syria and Turkey). Among the institutions with the highest number of publications in the DPJO are: University of São Paulo - Bauru Dental School (FOB-USP), Fluminense Federal University (UFF), São Paulo State University (UNESP - Araraquara), Federal University of Rio de Janeiro (UFRJ), Tehran University of Medical Science (Iran), Federal University of Bahia (UFBA), Federal University of Minas Gerais (UFMG), Federal University of Pará (UFPA), Pontifical Catholic University of Rio Grande do Sul (PUC-RS) and Rio de Janeiro State University (UERJ). They are renowned institutions in the labor of training postgraduates and/or graduates, which relied in the seriousness and quality of our journal.

It is time to thank those who, besides the many daily tasks, dedicate their time to the carving of the manuscripts published in this scientific journal. This citizenship work generates manuscripts with better reliability and a clear text. Among hundreds of researchers, we highlight ten with the highest number of peer-reviews completed in 2016 and 2017. They are: Marcio Almeida (UNOPAR), Emanuel Braga (UFBA), Daniela Feu (UVV), Lucas Abreu (UFMG), Célia Pinzan-Vercelino (UNICEUMA), Alex Pereira (UFMA), Hugo Caracas (DF), Luis Aidar (UNISANTA), Sergei Caldas (UFRN) and Ana Claudia Conti (USC). Thank you very much.

Now I leave, happy for completing this cycle and certain that every effort will be continued by this person I learned to truly admire in the last two years. A strong, but sensitive woman with clear and clever positions. A rare commitment to our specialty. Welcome, Prof Flavia Artese, your presence makes us contemplate a happy and fearless future.

David Normando - editor-in-chief (davidnormando@hotmail.com)

## Footnotes

* CAPES = Coordination for the Improvement of Higher Education Personnel.
